# An Over-Actuated Hexacopter Tilt-Rotor UAV Prototype for Agriculture of Precision: Modeling and Control

**DOI:** 10.3390/s25020479

**Published:** 2025-01-15

**Authors:** Gabriel Oliveira Pimentel, Murillo Ferreira dos Santos, José Lima, Paolo Mercorelli, Fernanda Mara Fernandes

**Affiliations:** 1 Department of Electroelectronics, Federal Center of Technological Education of Minas Gerais (CEFET-MG), Leopoldina 36700-001, Brazil; gabriel.pimentel@aluno.cefetmg.br; 2 Research Centre in Digitalization and Intelligent Robotics (CeDRI), Sustainability and Technology in Mountain Regions (SusTEC), Instituto Politécnico de Bragança (IPB), 5300-253 Bragança, Portugal; jllima@ipb.pt; 3 Institute for Production Technology and Systems (IPTS), Leuphana Universität Lüneburg, 21335 Lüneburg, Germany; paolo.mercorelli@leuphana.de; 4 Faculty of Minas Gerais (FAMINAS), Muriaé 36888-233, Brazil; fernandauss@hotmail.com

**Keywords:** hexacopter, over-actuated UAV, tilt-rotor

## Abstract

This paper focuses on the modeling, control, and simulation of an over-actuated hexacopter tilt-rotor (HTR). This configuration implies that two of the six actuators are independently tilted using servomotors, which provide high maneuverability and reliability. This approach is predicted to maintain zero pitch throughout the trajectory and is expected to improve the aircraft’s steering accuracy. This arrangement is particularly beneficial for precision agriculture (PA) applications where accurate monitoring and management of crops are critical. The enhanced maneuverability allows for precise navigation in complex vineyard environments, enabling the unmanned aerial vehicle (UAV) to perform tasks such as aerial imaging and crop health monitoring. The employed control architecture consists of cascaded proportional (P)-proportional, integral and derivative (PID) controllers using the successive loop closure (SLC) method on the five controlled degrees of freedom (DoFs). Simulated results using Gazebo demonstrate that the HTR achieves stability and maneuverability throughout the flight path, significantly improving precision agriculture practices. Furthermore, a comparison of the HTR with a traditional hexacopter validates the proposed approach.

## 1. Introduction

By definition, an unmanned aerial vehicle (UAV) is characterized by the absence of human presence on board its structure. In recent years, its use is currently growing, boosted by its ability to access remote or dangerous areas, facilitate surveillance, and capture high-resolution images, as well as the emergence of innovative computer technologies such as artificial intelligence (AI) and computer vision (CV), mainly in surveillance, terrestrial mapping, and the military field [1,2].

Initially inspired by radio-controlled model airplanes, most UAV applications were originally motivated by military purposes for operation in extreme conditions, allowing flights at lower altitudes with greater maneuverability to carry out challenging and dangerous tasks, such as surveillance, geographic reconnaissance, unmanned inspection, communications interception, and mapping enemy areas [3]. However, military use drove the increase in applications, resulting in its expansion into civilian use [4]. With technological advances in the civilian sector, UAVs (especially with the miniaturization of components, the evolution of navigation systems, and the integration of sophisticated sensors) have been widely used for various purposes, such as aerial photography [5], power line inspection [6,7], pollution monitoring [8], forest fire monitoring [9], rescue operations [10], disaster management [11], healthcare [12], traffic management [13], marine monitoring [14], and agricultural applications [15]. In other words, they are used in four major fields: environmental, security, communication, and monitoring applications.

In this context, agriculture has emerged as one of the sectors that most benefit from UAV technology [16]. Precision agriculture has emerged as a key approach with the growing need to optimize the production of agricultural and livestock products while minimizing waste and costs. It aims to increase the efficiency of agricultural operations through more precise monitoring and intervention, allowing farmers to manage their crops more effectively and sustainably, using advanced technologies. They can perform several tasks, including monitoring soil health, fertilizer application, and weather analysis [17,18].

Regarding UAV topologies, they are classified into three main categories: fixed wing (such as airplanes, which have high cruising speed and autonomous operating capacity), rotary wing (such as helicopters and multi-rotors, which have high maneuverability), and hybrid (such as tilt rotors, balloons, and airships, which have high autonomy but are slower) [19].

A notable configuration among UAVs are tilt rotors. They harmonize rotary wing characteristics with fixed wings, providing the capacity for vertical takeoffs and landings [20,21]. This gives them characteristics like helicopters, and fast and efficient horizontal flights, like airplanes. The over-actuated tilt rotors enable swift reactions to commands when in flight. They are found in various designs, which differ according to the number and arrangement of rotors in their structure. The most common topologies include quadcopters with four rotors and hexacopters with six rotors. The diversity of existing tilt rotors offers a wide range of advantages in terms of performance, maneuverability, and operational efficiency [22].

### 1.1. State of the Art and Related Works

Over the past few years, there have been significant remarkable advances in agriculture, most notably with the introduction of PA. This evolving field introduced innovative ways to improve crop productivity. It incorporates advanced technologies such as the global positioning system (GPS), sensors, and data analysis, allowing farmers to make more informed decisions and manage their land with greater accuracy [23]. Since its adoption, PA has shown considerable potential to revolutionize conventional farming practices, including crop monitoring and mapping.

With the ability to quickly inspect large areas, UAVs monitor crops more accurately, regularly, and cost-effectively, providing high-quality data and enabling wasteful or unproductive practices to be identified and eliminated. These vehicles can fly at specific altitudes and angles, ensuring detailed and consistent images. Moreover, UAVs offer a continuous record of crop health over time, valuable for analysis, research, and even insurance claims in the event of losses due to disease or extreme weather conditions. Another key advantage is their capacity to access areas that are difficult to access by traditional methods, such as mountainous terrain, dense vegetation, or large fields, allowing comprehensive monitoring over the entire agriculture field [24].

By incorporating the UAVs in their operations, producers have the advantage of receiving frequent updates and being instantly informed about the health of the crop within pre-defined limits. This allows them to identify areas that need intervention accurately. With the wide range of actionable data available, decisions can be made based on scientific evidence.

Given this promising scenario, several studies have explored using UAVs in precision agriculture. Meng and Cheng [25] explored the application of an octocopter for soil monitoring. The study integrates modified remote sensing data to accurately estimate soil available nutrients (SAN) at the field scale. Moreover, their UAV improved the accuracy of SAN estimation at the subfield level. The results demonstrated the potential of UAVs in soil monitoring, providing a more accurate tool to optimize fertilization strategies and support sustainable agricultural practices.

In studies focusing on yield estimation with a quadcopter, Apolo-Apolo et al. [26] developed a system to automate image processing methodology for citrus yield estimation, employing the quadcopter images and deep learning techniques. The results demonstrated the potential use of UAV for estimation. In another study, Feng et al. [27] equipped a UAV with infrared thermal cameras to estimate cotton yield by analyzing images from different stages of growth. The study concluded that images from UAVs are highly effective for estimating cotton yield, particularly when they are captured during critical growth stages such as flowering.

The research detailed by Shi et al. [28] investigates the efficacy of UAVs for pesticide application in tobacco pest management, focusing on how varying flight parameters affect spray performance. Conducting three field experiments at different stages of tobacco growth, from rosette to maturation, the researchers systematically assessed the effects of flight height, speed, and application volume on spray efficacy. The study showed that UAV spraying parameters are essential to improve pest control precision and efficiency.

Furthermore, in the work presented by Guo et al. [29], the use of a quadcopter equipped with hyperspectral sensors was explored to monitor wheat yellow rust at field scale. The research involved capturing hyperspectral images and analyzing various features (including vegetation indices and texture features) to develop partial least-squares regression models for disease monitoring during different infection stages. The study results concluded that UAV hyperspectral images are highly effective in monitoring wheat yellow rust, enabling valuable insights for disease management and early detection.

In addition, viticulture (a field of PA where monitoring vine health and yield is essential for vineyard management) has greatly benefited from adopting UAVs. Authors López-Granados et al. [30] equipped a quadcopter model MD4-1000 with a digital camera was used to acquire high-resolution images from four vineyards, generating point clouds to analyze each vine and measure woody crop canopies. Two approaches were tested: the first aimed to detect canopy management by comparing vine dimensions, and the second focused on determining whether post-treatment data could identify the management operations. The results demonstrated the success of the first approach, where canopy management interventions were effectively detected. In the second approach, significant differences in vine dimensions were observed after the treatments, and shoot trimming was easily and accurately identified. This study shows the capability of UAV technology combined with automated image analysis for precise monitoring.

Another significant study by Ferro et al. [31] compared different computer vision methods for detecting vine canopies using multispectral images captured by a quadcopter. They showed that the multispectral image analysis technologies captured by UAVs represent an additional asset for the comprehensive monitoring and prediction of vineyard vegetation conditions. Moreover, Pádua et al. [32] explored vineyard classification using the OBIA (object-based image analysis) technique on data obtained by two UAVs: a quadcopter and a hexacopter. The results presented that data acquired from different sensors are suitable to be used in the vineyard classification process.

Unfortunately, considering over-actuated UAVs, there is a notable lack of research directly focused on their application to precision agriculture. Most studies focus on the general use of UAVs in PA with conventional configurations. However, some studies suggest potential applications for over-actuated UAVs. A recent study from UC Berkeley [33] explores the design and control of an over-actuated quadcopter that improves control and stability, particularly in difficult flight conditions. Although this research is not directly focused on agriculture, the presented results contribute to advancing UAV technologies with potential applications in PA.

Over-actuated topologies are particularly advantageous for PA due to their enhanced maneuverability and increased payload capacity. These configurations enable the optimization of tilt angles, reducing aerodynamic drag and improving overall energy efficiency. Also, multirotors can offer vertical taking off and landing. In addition, the redundancy provided by these systems enhances flexibility, enabling the implementation of advanced control strategies that improve performance, fault tolerance, and reliability [34].

### 1.2. Contributions

Taking this scenario into consideration, this paper aims to present the modeling and control of a new over-actuated hexacopter tilt-rotor with all eight actuators operating independently for precision agriculture applications.

This design offers significant advantages over previously discussed projects, particularly due to the configuration where two of the six propulsion motors are independently tilted by the servomotors. This setup enhances the UAV’s maneuverability and flight reliability. To achieve this, an extended fast control allocation (FCA) method is implemented, which divides the control effectiveness matrix (CEM) into two subsystems, fully superposed, allowing a fast and decoupled control allocation technique to embed in control boards with low capability effort. The SLC was also implemented to integrate the respective control loops.

**Figure 1:**
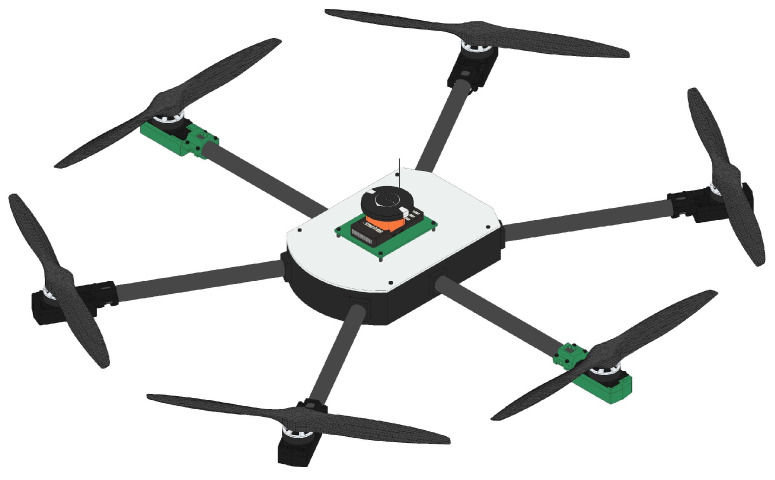
Proposed HTR with two servomotors in upward direction.

**Figure 2:**
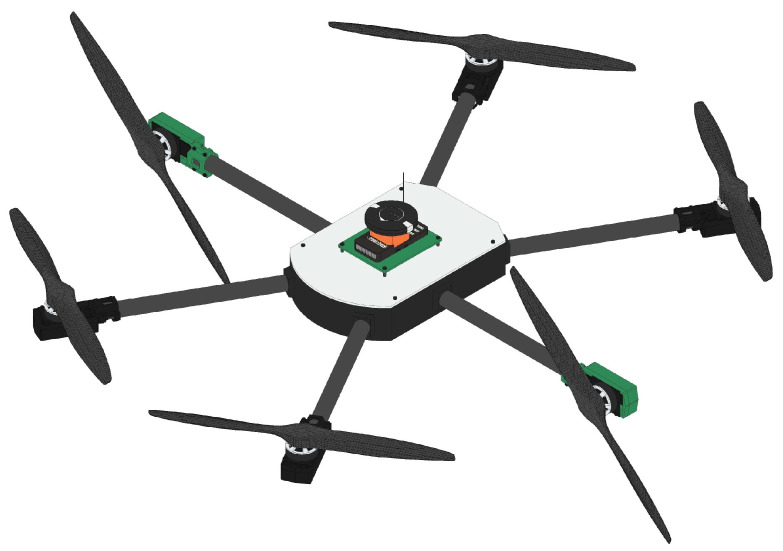
Proposed HTR with two servomotors tilted 90°.

Figure 1 and Figure 2 show the over-actuated HTR, with emphasis on the inclination of the two tilt rotors of the aircraft. Given this configuration, it has high maneuverability in forward dynamics considering the tilt of the motors, rather than pitching the aircraft down/up. Moreover, the HTR has a high-speed capability for minor trajectory adjustments and this configuration allows for faster yaw maneuvers if the tilt rotors are tilted in opposite directions:

In addition, by maintaining 90 degrees of pitch angle, the two tiltable motors are positioned horizontally. This particularity of tilt-rotor UAVs, known for their ability to combine rotary-wing capabilities with the efficient forward-flight characteristics of fixed-wing aircraft, as demonstrated by Rojo-Rodriguez et al. [35], enhances flight efficiency. In other words, the tilt rotors allow the HTR to move forward or backward without the need to change the pitch angle, resulting in a more energy-efficient flight, as the UAV expends less energy on drag forces.

Focusing on over-actuated UAVs, this work is an extension of previous research in this area. A study by Leal Lopes et al. [7] explored the design of an over-actuated HTR for landing and coupling in power transmission lines, but with a different FCA extension. Additionally, a previous study by Santos et al. [36] focused on the analysis of FCA applied for nonlinear over-actuated systems. They provided an investigation into control allocation techniques that enhance the maneuverability and stability of over-actuated systems.

Although these studies did not directly focus on precision agriculture or exclusively on two tilting propulsion motors, they served as a basis for over-actuated HTR control. Furthermore, this work is a continuation of the research previously presented by Pimentel et al. [37] which focused only on MATLAB simulations to validate the control and modeling of the HTR. This paper extends that work by adopting a more comprehensive approach, using software-in-the-loop simulations in Gazebo to replicate PA scenarios with real-world coordinates. Moreover, the control algorithms are implemented directly into the PX4 firmware, demonstrating practical applicability. This study also evaluates the performance of the HTR in complex agricultural missions, showcasing its suitability for real-world PA applications.

Taking this into consideration, the main contributions of this work are highlighted below:

- Modeling and control of a new over-actuated HTR prototype, equipped with eight independently operating actuators, designed specifically for PA applications;
- Description of the two propulsion motors, which can be independently adjusted using servomotors, enabling enhanced maneuverability, forward and backward movements without the need to change the pitch angle, and more efficient yaw control;
- Simulations using software-in-the-loop in the Gazebo software environment using real agricultural scenarios, allowing the evaluation of performance in complex PA missions and validating the aircraft in close to real-world conditions before experimental tests with the HTR’s physical implementation;
- Implementation of an extended FCA technique within the PX4 board, which divides the CEM into two subsystems, allowing fast and decoupled control allocation;
- Implementation of the control structure with a cascaded P- PID topology directly into the PX4 firmware, making the HTR able to operate in physical environments and perform missions in real PA scenarios;
- Development of a routine that enables the connection of servomotors and propulsion motors (devices with different pulse width modulation (PWM) frequencies), directly influencing the FCA convergence procedure.

**Figure 3:**
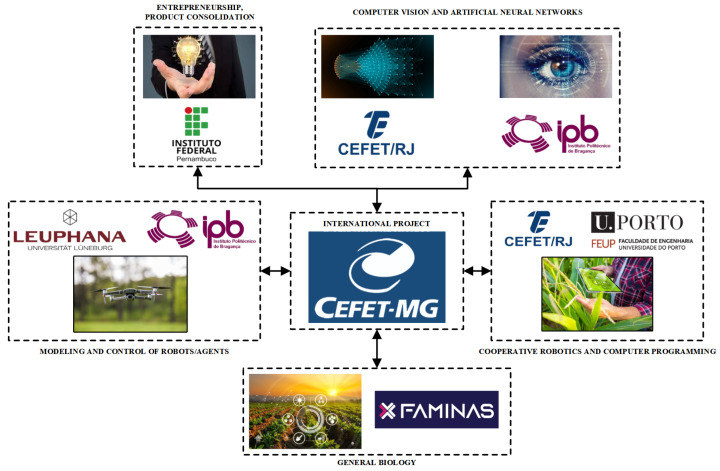
Illustration of the international cooperation universities and their respective concentration areas.

Furthermore, the HTR significantly contributes to an international cooperation project titled Study of Cooperative and Autonomous Inspection in Plantations, which was founded by the National Council for Scientific and Technological Development (CNPq) and is registered under number 442696/2023-0. Its main goal is developing an innovative cooperative system involving robots and other autonomous agents to inspect fields using computer vision techniques, sensor integration, and intelligent cooperation. With its unique features, such as enhanced maneuverability, energy-efficient flight, and rapid yaw maneuvers, the HTR enables precise and efficient navigation in agricultural environments, thereby optimizing field inspection processes. Figure 3 illustrates it in the whole project, where this work falls within the Modeling and Control of Robots/Agents area:

This project involves members from seven academic institutions from three different countries: CEFET-MG (Federal Center for Technological Education of Minas Gerais), CEFET/RJ (Federal Center for Technological Education Celso Suckow da Fonseca), IFPE (Federal Institute of Pernambuco), and FAMINAS (Faculty of Minas Gerais) from Brazil; Leuphana University of Lüneburg from Germany; and IPB (Polytechnic Institute of Bragança), and FEUP (Faculty of Engineering of the University of Porto) from Portugal.

In this collaboration, CEFET-MG serves as the executing institution, playing a central role in all aspects of the project. Its responsibilities include mission planning, establishing communication between the ground station and robots using robot operating systems (ROSs) and MAVLink protocols, and overseeing overall project coordination. Additionally, CEFET-MG team members conduct simulations in the Gazebo environment to validate methodologies prior to open-field experimental tests, ensuring the integration and effectiveness of the proposed solutions.

Leuphana University and the Polytechnic Institute of Bragança take the lead in robot modeling and control, developing advanced controllers and implementing control allocation techniques for all agents, with support from CEFET-MG members. Furthermore, IPB members, in collaboration with CEFET/RJ and FEUP, focus on applying CV techniques and artificial neural networks (ANNs) for detecting agricultural challenges such as irrigation deficiencies and pest infestations. Meanwhile, FAMINAS contributes essential biological insights by analyzing visual patterns in plantations to inform the development of ANN-based detection models. Finally, IFPB team members focus on product consolidation, conducting feasibility studies to ensure the project’s economic and technical viability, aligning its outcomes with market demands.

### 1.3. Paper Organization

The paper is structured as follows: Section 2 presents the kinematics and dynamics of the HTR modeling. Section 3 presents the overall developed control structure; Section 4 depicts the FCA technique applied to the HTR. Section 5 provides an overview of the simulation environment used and the results by Gazebo environment. Finally, Section 6 concludes this work and suggests future works.

## 2. HTR Kinematics and Dynamics Modeling

The objective of this section is to present the definitions and basic concepts that describe the modeling of the kinematics and dynamics of the HTR.

**Figure 4:**
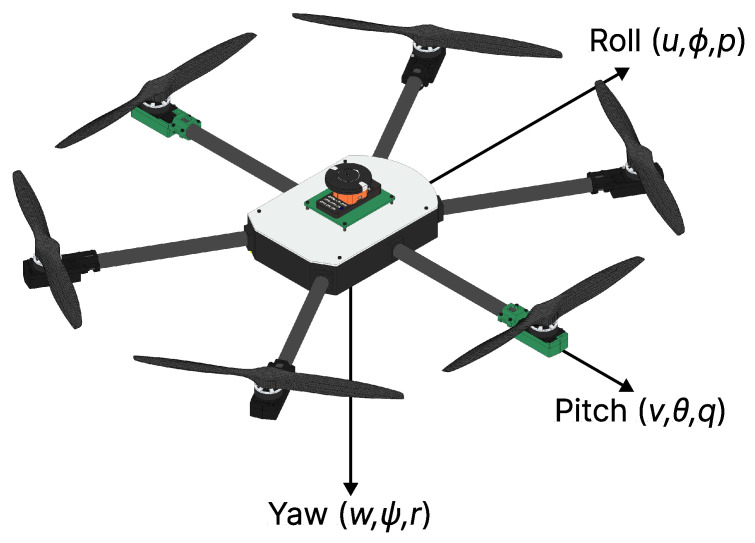
HTR with its state variables.

The aircraft variables are measured on the vehicle frame $F^{v}$, the fixed body frame $F^{b}$, and the inertia frame $F^{i}$. From this, UAVs have six DoFs that influence their movements, including translations in the *x*, *y*, and *z* axes and rotations in the angles of roll, pitch, and yaw, used to describe the aircraft’s angular movements. For better understanding, Figure 4 shows the respective axes:

The variables ϕ, θ, and ψ represent the roll, pitch, and yaw angles, respectively. The variables *p*, *q*, and *r* represent the roll, pitch, and yaw rates, respectively. The variables *u*, *v*, and *w* correspond to the HTR velocities measured along their fixed frame axes.

Accordingly, Equations (1) and (2) describe the body frame inertial velocities (*u*, *v*, and *w*), and the inertial position ($p_{n}$, $p_{e}$, and −h) as follows:

(1) $$\frac{d}{dt}\left(\begin{array}{c} p_{n}\\ p_{e}\\ -h \end{array}\right)=R_{v}^{b}\left(\begin{array}{c} u\\ v\\ w \end{array}\right)={\left(R_{v}^{b}\right)}^{T}\left(\begin{array}{c} u\\ v\\ w \end{array}\right),$$

(2) $$R_{v}^{b}=\left(\begin{array}{ccc} c\theta c\psi & s\phi s\theta c\psi -c\phi s\psi & c\phi s\theta c\psi +s\phi s\psi \\ c\theta s\psi & s\phi s\theta s\psi +c\phi c\psi & c\phi s\theta s\psi -s\phi c\psi \\ -s\theta & s\phi c\theta & c\phi c\theta \end{array}\right).$$

The relationship between the angles of roll, pitch, and yaw (ϕ, θ, ψ) and their corresponding angular rates (*p*, *q*, *r*) must also be considered for frame transformations [38]. The angular rates are defined in $F^{b}$, the roll angle is referenced in $F^{v2}$, the pitch angle in $F^{v1}$, and the yaw angle in $F^{b}$, for which it is necessary to use rotational matrix [39], resulting in Equation (3):

(3) $$\left(\begin{array}{c} p\\ q\\ r \end{array}\right)=\left(\begin{array}{ccc} 1 & 0 & -s\theta \\ 0 & c\phi & s\phi c\theta \\ 0 & -s\phi & c\phi c\theta \end{array}\right)\left(\begin{array}{c} \dot{\phi }\\ \dot{\theta }\\ \dot{\psi } \end{array}\right).$$

Manipulating the Equation (3), is given the Equation (4), as follows:

(4) $$\left(\begin{array}{c} \dot{\phi }\\ \dot{\theta }\\ \dot{\psi } \end{array}\right)=\left(\begin{array}{ccc} 1 & s\theta t\theta & c\phi t\theta \\ 0 & c\phi & -s\phi \\ 0 & s\phi /c\theta & c\phi /c\theta \end{array}\right)\left(\begin{array}{c} p\\ q\\ r \end{array}\right).$$

The dynamic modeling of a hexacopter is made using translational and rotational movements, applying Newton’s second law [38,40]. Taking *v* as the HTR velocity in the inertial frame $F^{i}$, applying Newton’s law to translational motion yields:

(5) $$f=m\frac{dv}{dt_{i}}.$$

where *m* is the HTR total mass, and f is the total force applied to the vehicle. So, from equation of Coriolis, Equation (5) can be reformulated into:

(6) $$f=m\frac{dv}{dt_{i}}=m\left(\frac{dv}{dt_{b}}+\omega_{b/i}\times v\right).$$

Given that the control force is measured and applied in the body frame $F^{b}$, and considering that the angular velocity $\omega_{b/i}$ is also measured in the body frame, Equation (6) can be rewritten in the body reference frame. Here, $v^{b}≜{\left(u,v,w\right)}^{T}$, and $\omega_{b/i}^{b}≜{\left(p,q,r\right)}^{T}$. Consequently, in the body frame, it can be expressed as follows [38]:

(7) $$\left(\begin{array}{c} \dot{u}\\ \dot{v}\\ \dot{w} \end{array}\right)=\left(\begin{array}{c} rv-qw\\ pw-ru\\ qu-pv \end{array}\right)+\frac{1}{m}\left(\begin{array}{c} X_{p}^{b}\\ Y_{p}^{b}\\ Z_{p}^{b} \end{array}\right),$$

where $X_{p}^{b}$, $Y_{p}^{b}$ and $Z_{p}^{b}$ are the resulting forces for rolling, pitching, and yawing in the frame $F^{b}$.

For rotational motion, Newton’s second law becomes:

(8) $$\frac{dh^{b}}{dt_{i}}=t_{b},$$

where $h^{b}\in R^{3}$ represents the angular momentum of the body, and $t_{b}\in R^{3}$ denotes the sum of all applied torques. This expression is true for applied moments at the center of mass [38].

Applying again the equation of Coriolis:

(9) $$\frac{dh}{dt_{i}}=\frac{dh}{dt_{b}}+\omega_{b/i}\times h=t_{b}.$$

In rigid bodies, angular momentum is defined as the product of the inertia matrix J and the angular velocity vector, represented as $h^{b}≜J\omega_{b/i}^{b}$ [41]. The matrix J is expressed as follows:

(10) $$J≜\left(\begin{array}{ccc} J_{x} & -J_{xy} & -J_{xz}\\ -J_{xy} & J_{y} & -J_{yz}\\ -J_{xz} & -J_{yz} & J_{z} \end{array}\right).$$

Considering the HTR is symmetric about the coordinate axes (${\hat{i}}^{b}$, ${\hat{j}}^{b}$, and ${\hat{k}}^{b}$), it is implied that the moments of inertia $J_{xz}=J_{xy}=J_{yz}=0$. So, Equation (9) can be transformed into:

(11) $$J^{b}\frac{d\omega_{b/i}}{dt_{b}}+\omega_{b/i}\times J^{b}\omega_{b/i}=t_{b}.$$

From this, the angular velocity can be described using the derivative matrix of the instantaneous projections on the respective axes in the fixed body frame $F^{b}$ corresponding to the roll, pitch, and yaw rates (*p*, *q*, and *r*). It is obtained by isolating the angular velocity from Equation (11):

(12) $$\left(\begin{array}{c} \dot{p}\\ \dot{q}\\ \dot{r} \end{array}\right)=J^{b^{-1}}\left(\begin{array}{c} -\left[\begin{array}{c} \dot{p}\\ \dot{q}\\ \dot{r} \end{array}\right]\times J^{b}\left[\begin{array}{c} \dot{p}\\ \dot{q}\\ \dot{r} \end{array}\right]+\left[\begin{array}{c} L_{p}^{b}\\ M_{p}^{b}\\ N_{p}^{b} \end{array}\right] \end{array}\right).$$

where $L_{p}^{b}$, $M_{p}^{b}$, and $N_{p}^{b}$ are the resultant torques for rolling, pitching and yawing, respectively, in the fixed body frame $F^{b}$.

Furthermore, the six DoF model for the HTR kinematics and dynamics can be summarized by Equations (1), (4), (7), and (12).

**Figure 5:**
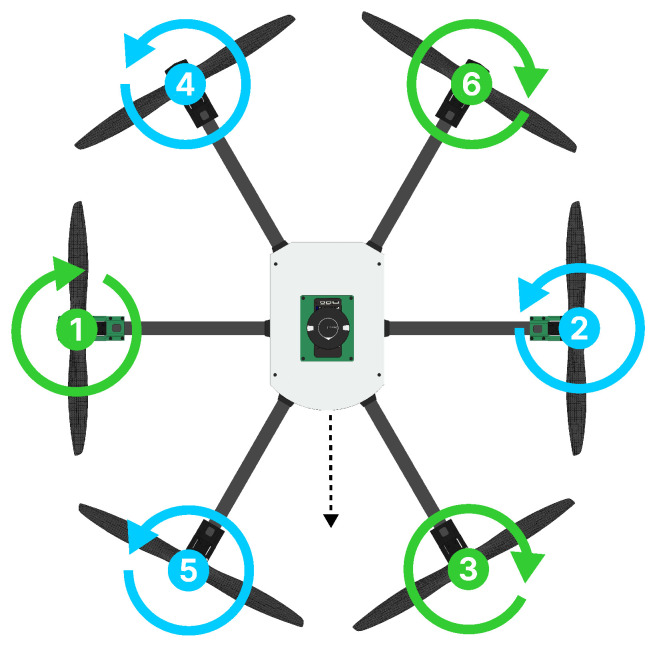
HTR with propulsion motors and respective rotation directions.

However, to better comprehend the forces and torques produced by the six HTR operating motors, it is crucial to understand the directions of rotation for each motor, as these influence the propulsive forces and angular moments. Then, the propulsion motors with their respective directions of rotation are shown in Figure 5:

Rotors 1, 3, and 6 (indicated in green ) rotate in a clockwise direction, while rotors 2, 4, and 5 (indicated in blue spin) rotate in a counter-clockwise direction. Furthermore, rotors 1 and 2 are the only two propulsion motors that can be independently tilted by the servomotors. To move the HTR in the forward direction (relative to the aircraft front), tilt rotors 1 and 2 move at the same positive angle. Negative angles for rotors 1 and 2 displace the HTR in the backward direction. Moreover, with opposing angles in tilt rotors, the HTR performs yaw maneuvers.

Therefore, the resultant forces produced by the motors in vehicle body frame $F^{b}$ along axis $i^{b}$, $j^{b}$ are divided into propulsion ($X_{p}^{b}$, $Y_{p}^{b}$, $Z_{p}^{b}$), aerodynamic ($X_{a}^{b}$, $Y_{a}^{b}$, $Z_{a}^{b}$), and gravity ($X_{g}^{b}$, $Y_{g}^{b}$, $Z_{g}^{b}$) forces, given by Equation (13):

(13) $$\left(\begin{array}{c} X^{b}\\ Y^{b}\\ Z^{b} \end{array}\right)=\left(\begin{array}{c} X_{p}^{b}\\ Y_{p}^{b}\\ Z_{p}^{b} \end{array}\right)+\left(\begin{array}{c} X_{a}^{b}\\ Y_{a}^{b}\\ Z_{a}^{b} \end{array}\right)+\left(\begin{array}{c} X_{g}^{b}\\ Y_{g}^{b}\\ Z_{g}^{b} \end{array}\right).$$

Considering the right-hand rule and the HTR particularities, the $Y_{p}^{b}$ propulsion force along the $j^{b}$ axis was disregarded (0 N).

Moreover, the resulting torques in the HTR are divided into propulsion ($L_{p}^{b}$, $M_{p}^{b}$, $N_{p}^{b}$), aerodynamic ($L_{a}^{b}$, $M_{a}^{b}$, $N_{a}^{b}$) and gravity ($L_{g}^{b}$, $M_{g}^{b}$, $N_{g}^{b}$) torques, given by Equation (14):

(14) $$\left(\begin{array}{c} L^{b}\\ M^{b}\\ N^{b} \end{array}\right)=\left(\begin{array}{c} L_{p}^{b}\\ M_{p}^{b}\\ N_{p}^{b} \end{array}\right)+\left(\begin{array}{c} L_{a}^{b}\\ M_{a}^{b}\\ N_{a}^{b} \end{array}\right)+\left(\begin{array}{c} L_{g}^{b}\\ M_{g}^{b}\\ N_{g}^{b} \end{array}\right).$$

From the forces and torques, gravitational and aerodynamic forces are inherent to the HTR’s characteristics; thus, the virtual control actions (VCAs) are performed only by the propulsion forces and torques, which are directly related to real control actions (RCAs) [41]. Consequently, the HTR CEM is:

(15) $$\left(\begin{array}{c} X_{p}^{b}\\ Z_{p}^{b}\\ L_{p}^{b}\\ M_{p}^{b}\\ N_{p}^{b} \end{array}\right)=\left(\begin{array}{c} k_{1}s\gamma_{1}\delta_{1}+k_{1}s\gamma_{2}\delta_{2}\\ k_{1}c\gamma_{1}\delta_{1}+k_{1}c\gamma_{2}\delta_{2}+k_{1}\left(\delta_{3}+\delta_{4}+\delta_{5}+\delta_{6}\right)\\ \left(k_{1}c\gamma_{1}\ell -k_{2}s\gamma_{1}\right)\delta_{1}+\left(-k_{1}c\gamma_{2}\ell +k_{2}s\gamma_{2}\right)\delta_{2}+k_{1}\ell \frac{1}{2}\left(\delta_{3}-\delta_{4}-\delta_{5}+\delta_{6}\right)\\ k_{1}\ell \frac{\sqrt{3}}{2}\left(\delta_{3}-\delta_{4}+\delta_{5}-\delta_{6}\right)\\ \left(-k_{1}s\gamma_{1}\ell -k_{2}c\gamma_{1}\right)\delta_{1}+\left(k_{1}s\gamma_{2}\ell +k_{2}c\gamma_{2}\right)\delta_{2}+k_{2}\left(-\delta_{3}+\delta_{4}+\delta_{5}-\delta_{6}\right) \end{array}\right),$$

where ℓ=0.25 m is the arm length between the respective propeller and the HTR center of gravity (CG), $k_{1}=7.81$ N and $k_{2}=0.00781$ Nm are constants related to propulsion forces and torques, $\delta_{*}$ is the command signal for each motor, $\gamma_{*}$ is the tilt rotor angle of each servomotor, and $s\gamma_{j}=\sin\gamma_{j}$ and $c\gamma_{j}=\cos\gamma_{j}$.

## 3. Control Structure

This section describes the overall control structure applied to the over-actuated HTR, which is conducted at the upper levels of the control allocation.

For the successful development of UAVs, several steps are crucial, such as comparing mathematical modeling and the real system, as well as the implementation of control methods to ensure stability and safety in extreme operating situations, including mechanical failures [42,43]. This allows the vehicle to operate smoothly and harmoniously, and for this, it is important to use the control technique to operate in an agile and structured procedure [44].

Achieving an aircraft with adequate stability and control characteristics is a fundamental step. To achieve this objective, specific requirements must be satisfied. The essential characteristics that the vehicle must provide to perform its functions safely and efficiently are as follows:

- Sufficient controllability to sustain level flight and transition from one equilibrium condition to another;
- The control forces must be within the permissible limits provided in the design;
- The airplane must be capable of stabilization throughout the flight envelope.

Several control techniques are currently recognized, including P and PID controllers. Among these approaches, the choice of the most appropriate technique depends on several factors, such as the characteristics of the aircraft, its complexity, the operating environment, and performance requirements. Although other alternatives are available, PID control is widely used due to its effectiveness, low computational cost, and continued relevance in the UAV field [45].

Taking this into consideration, Basri et al. [46] investigated the quadcopter trajectory tracking using a PID controller. Their study presented the mathematical modeling of the quadcopter using Newton–Euler equations. Then, a manually tuned PID controller was designed to ensure stable flights and effective operation. Finally, the quadcopter was tested on five distinct trajectories, with the results demonstrating that the controller consistently and accurately tracked the desired flight paths.

In another related work, Rajan et al. [47] presented the control of a hexacopter sprayer for deforestation and pest control. In this study, a PID controller was implemented, and sensor performance was evaluated to ensure the proper functioning of the hexacopter. A comparison with traditional methods revealed that the hexacopter sprayer outperformed conventional approaches in both efficiency and effectiveness. The UAV covered more ground in less time and was able to access challenging areas, reducing the need for manual labor. The PID controller contributed to stable altitude maintenance and effective obstacle avoidance throughout the process.

Despite the popularity of classical controllers, the increased demand for higher accuracy and robustness in dynamic environments has stimulated the development of sophisticated control methods. Chiew et al. [48] applied a second-order sliding mode control (SMC) for altitude and yaw quadcopter tracking control and compared it to a traditional PID controller. Two types of input were tested: a slow rate input with a single setpoint (SP) and a fast rate input with multiple SPs. Simulation results demonstrated that the SMC controller approach consistently outperformed the traditional PID across all performance metrics.

Furthermore, Al-Mahasneh et al. [49] proposed an adaptive neural network (NN) controller for altitude tracking and attitude stabilization of a hexacopter with uncertain dynamics. The design, simulation, and robustness of the controller against gust disturbances were analyzed. Furthermore, the controller performance was compared with a standard filtered PID controller across different control scenarios. The results showed that the adaptive NN controller demonstrated quick and highly accurate performance controlling altitude and attitude. Additionally, this controller outperformed the filtered PID in multiple scenarios, providing better adaptation capabilities and reduced overshoot.

**Figure 6:**
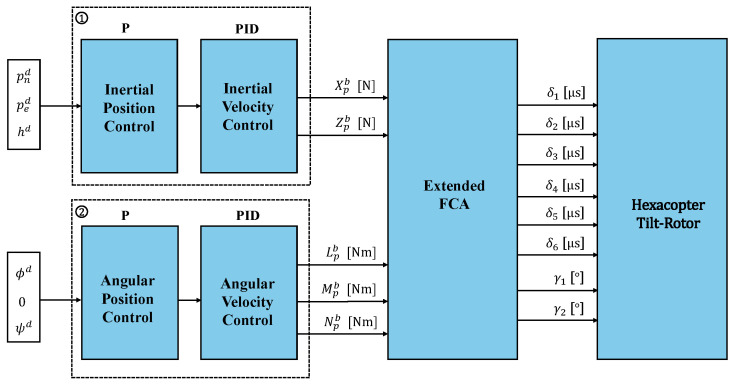
Illustration of the HTR full control loops.

Another notable controller configuration, which is employed in this paper, is cascade P-PID topology. This controller is implemented considering two cascade levels: external and internal. The external uses a P controller and the internal uses a PID controller, so the output of the external control action is the input of the internal cascade controller, refining the dynamic response to ensure precision and robustness in the control [50]. Figure 6 shows the control structure schematically:

Considering the figure, the upper-level control structure includes position and velocity inertial controllers, and was implemented using two levels of cascaded closed-loop control: an external and an internal level. The external level includes a proportional controller for inertial position, while the internal level consists of a PID controller for inertial velocity.

The external controller is responsible for maintaining the inertial position, specifically controlling altitude (*h*) and the north and east positions, ($p_{n}$) and ($p_{e}$), respectively. The P controller was adopted for its capability of fast response to changes and its efficiency, while the PID controller was adopted for its easy implementation. Moreover, the position and velocity inertial loop operates at a rate of 50 Hz.

In this upper level, the P controller manages the inertial position, and its output is the input to the subsequent PID controller, which manages the inertial velocity. Two outputs from this control level, $X_{p}^{b}$ and $Z_{p}^{b}$, represent the required forces to be applied to the CEM to generate the control actions. In addition, this upper-level generates SPs for roll and yaw based on the east ($p_{e}$) and north ($p_{n}$) positions using the line of sight (LoS) method. This ensures the HTR follows the desired trajectory by calculating the appropriate orientation ($\phi^{d}$ and $\psi^{d}$) to align the aircraft with the target path and ensures precise path following.

Since direct lateral force control is unavailable in this configuration, lateral motion is achieved indirectly by generating a roll SP. Simultaneously, the desired heading (yaw SP) is derived from east and north position SPs, ensuring correct orientation throughout the aircraft’s movements.

At the lower level, the angular position and velocity controllers are illustrated, which perform a fundamental function in the control of angular stability through roll (ϕ), pitch (θ), and yaw (ψ) angles. Similarly to the upper level, two cascaded loops are implemented: an external and an internal loop. The external loop represents angular position control using a proportional controller, while the internal loop performs angular velocity control employing a PID controller. The angular position loop operates at a rate of 250 Hz, while the angular velocity loop operates at a rate of 1 kHz.

The outputs from this lower level, $L_{b}^{p}$, $M_{b}^{p}$, and $N_{b}^{p}$, represent the desired roll, pitch, and yaw torques in the body-fixed frame, which are then used in the HTR CEM to generate appropriate control actions. Moreover, it is important to note that the pitch SP is set to zero due to the unique hexacopter arrangement, where servomotors are responsible for tilting to compensate for pitch movements, ensuring stability and precision in the control system.

The controller tuning was performed based on the linear model, using the methodology proposed by Beard and McLain [38] and Santos et al. [51], ensuring a robust technique for the system performance. Accordingly, Table 1 summarizes the P-PID controller gains. These values, designed and optimized based on the discussed methodology, offer an effective solution for all five aircraft controlled DoFs.

### Servomotors and Propulsion Motors Interaction

The servomotors and propulsion motors in the aircraft introduce challenges due to the significant difference in their PWM frequencies. The propulsion motors operate at a frequency of 400 Hz (same as the inner attitude control loops) and the servomotors at 50 Hz. Therefore, within one actuation cycle of the servomotors, the propulsion motors undergo eight actuation cycles.

This discrepancy has a direct impact on the convergence of the extended FCA algorithm. This control allocation technique assumes that servomotors achieve the commanded positions. However, due to the lower update rate, the actual servo position delays the expected position for seven out of eight propulsion motor actuation cycles. Consequently, the FCA processes estimated servo positions during these cycles, resulting in inaccuracies in the control output, directly interfering in its process convergence.

**Figure 7:**
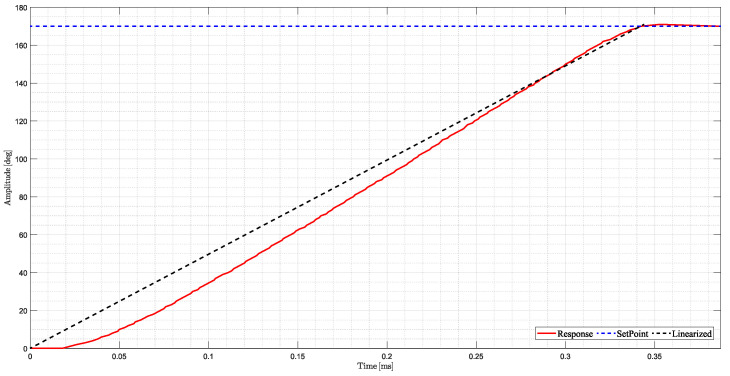
Illustration of the servomotor test bench experiments.

To address this issue, a routine was developed to manage the synchronization and interaction between the servomotors and the propulsion motors. This routine ensures that the FCA algorithm appropriately accounts for the slower update rate of the servomotors by refining position estimates and updating the actual servo position at appropriate intervals. Test bench experiments were done, where Figure 7 presents the linearized function:

This synchronization mechanism minimizes the impact of frequency offset on the control allocation process, ensuring improved convergence and stability in the HTR’s performance, particularly in PA scenarios, which require high precision and maneuverability [52,53].

## 4. Extended FCA Technique

This section focuses on the FCA technique, which is essential for optimizing control distribution among the over-actuated HTR actuators. By decomposing the control challenges into simpler components, the FCA enhances the efficiency and responsiveness of the control system.

A major challenge of nonlinear control strategies in unmanned systems is their high computational cost, which becomes particularly problematic for small, lightweight devices requiring real-time processing.

Generally, control allocation techniques are implemented through four primary methods: direct allocation [54], pseudo-inverse [55], linear programming [56], and nonlinear programming [57]. In this context, the fast control allocation method has emerged as a solution by reducing the complexity of nonlinear control through two parts: separation and mapping, allowing a more efficient and lightweight control allocation.

Furthermore, the FCA technique divides the RCAs into groups. This separation transforms the complex nonlinear problem into simpler linear subproblems. Then, mapping is applied to associate each VCA with its corresponding linear subproblem. This decomposition of the nonlinear control space into linear subspaces allows FCA, providing a computationally efficient control allocation, including in resource-limited environments.

The stability of the FCA method was confirmed through previous rigorous analysis, demonstrating that the solutions converge and remain within acceptable ranges throughout the control allocation process. The effectiveness of FCA is further evidenced by its ability to handle different system configurations, ensuring that the VCAs are accurately mapped to their corresponding linear subproblems. This approach guarantees robust and reliable system operations, even under challenging conditions with limited resources. The combination of stability, efficiency, and adaptability places FCA as a promising solution for over-actuated systems.

Additionally, the objective of the fast control allocation technique is to transform the nonlinear control allocation into a faster linear version, transforming VCA into RCA. So, the proposed extension is hereafter formulated. First, the nonlinear system is below:

(16) $$\hat{\tau }=M\left(u\right),$$

where M is the CEM presented in Equation (15), and $\hat{\tau }$ and u are the VCA and RCA vectors, respectively.

This control allocation method provides new linear spaces from a nonlinear space, allowing the nonlinear system to be broken into two different problems, as shown in Equations (17) and (18):

(17) $$\begin{array}{ccc} \hat{\tau_{a}} & = & {M_{a}\left(u_{b}\right)u_{a}}^{\prime }, \end{array}$$

(18) $$\begin{array}{ccc} \hat{\tau_{b}} & = & M_{b}\left(u_{a}\right)u_{b}, \end{array}$$

where $u_{a}\in R^{q}$, with $q\in N^{*}$ being a part of the *n* actuators of the system, $u_{b}\in R^{r}$, with $r\in N^{*}$ being the rest of the actuators, $\hat{\tau_{a}}\in R^{ma}$, $\hat{\tau_{b}}\in R^{mb}$ with $m\in N^{*}$ being the number of DoFs in RCA, $M_{a}\left(u_{a}\right)\in R^{ma\times q}$ and $M_{b}\left(u_{b}\right)\in R^{mb\times r}$.

To apply the FCA technique to the HTR and minimize the linearization process between nonlinear and linear expressions, the extended approach consists of separating these terms into distinct groups, fully superposed: nonlinear terms in $u_{a}$, and linear terms in $u_{b}$. These terms are represented by $\sin\gamma_{i}$ and $\delta_{i}$, respectively. From this, Equation (19) is obtained:

(19) $$u_{a}^{\prime }\in R^{3}={\left[\sin\left(\gamma_{1}\right),\sin\left(\gamma_{2}\right),1\right]}^{T},$$

In this formulation, the value 1 is responsible for the sum of all terms related to cos. This choice consolidates and allows nonlinearities to be represented as internal constants. The advantage of this method consists of acting as a normalization factor. Also, since $u_{a}$ is derived from the system, it allows adjustments to be made so that the value can deviate from 1 when necessary.

This normalization method allows the entire vector $u_{a}^{\prime }$ to be adjusted to ensure that the final term remains equal to 1. Equation (20) demonstrates how this adaption facilitates the use of RCA to operate the aircraft servomotors with genuine control signals:

(20) $$u_{a}\in R^{2}={\left[\sin^{-}\left(\gamma_{1}\right),\sin^{-}\left(\gamma_{2}\right)\right]}^{T}.$$

For the second group of RCA, the linear terms ($\delta_{i}$) representing the thruster rotations are organized by Equation (21):

(21) $$u_{b}\in R^{6}={\left[\delta_{1},\delta_{2},\delta_{3},\delta_{4},\delta_{5},\delta_{6}\right]}^{T}.$$

The next step in the design of the system is the selection of two groups of VCAs: $\tau_{a}$ and $\tau_{b}$. For this purpose, the configuration of the total VCA superposition between the two sets was adopted, as recommended by Santos et al. [36]. Consequently, $M_{a}\left(u_{a}\right)\in R^{2\times 6}$ and $M_{b}\left(u_{b}\right)\in R^{5\times 6}$ are described by the Equations (22) and (23):

(22) $$\begin{array}{ccc} {\hat{\tau }}_{a} & = & {\left[X_{p}^{b},N_{p}^{b}\right]}^{T}, \end{array}$$

(23) $$\begin{array}{ccc} {\hat{\tau }}_{b} & = & {\left[X_{p}^{b},Z_{p}^{b},L_{p}^{b},M_{p}^{b},N_{p}^{b}\right]}^{T}. \end{array}$$

The choice of terms $X_{p}^{b}$ and $N_{p}^{b}$ for the first virtual control action subgroup is justified by their role in stabilizing the vehicle orientation, given that they are directly controlled by the action set $u_{a}$. Subsequently, the remaining virtual variables are allocated to the group $u_{b}$. Equations (24) and (25) show the corresponding subgroups:

(24) $$\begin{array}{c} \hat{\tau_{b}}\\ \overset{︷}{\left[\begin{array}{c} X_{p}^{b}\\ N_{p}^{b} \end{array}\right]} \end{array}=\begin{array}{c} M_{a}\left(u_{a}^{-},u_{b}\right)\\ \overset{︷}{\left[\begin{array}{ccc} k_{1}\delta_{1} & k_{1}\delta_{2} & 0\\ -k_{1}l\delta_{1} & k_{1}l\delta_{2} & -k_{2}\delta_{3}+k_{2}\delta_{4}+k_{2}\delta_{5}-k_{2}\delta_{6}-k_{2}\cos\gamma_{1}\delta_{1}+k_{2}\cos\gamma_{2}\delta_{2} \end{array}\right]} \end{array}\begin{array}{c} {u_{a}}^{\prime }\\ \overset{︷}{\left[\begin{array}{c} \sin(\gamma_{1})\\ \sin(\gamma_{2})\\ 1 \end{array}\right]} \end{array},$$

(25) $$\begin{array}{c} \hat{\tau_{a}}\\ \overset{︷}{\left[\begin{array}{c} X_{p}^{b}\\ Z_{p}^{b}\\ L_{p}^{b}\\ M_{p}^{b}\\ N_{p}^{b} \end{array}\right]} \end{array}=\begin{array}{c} M_{b}\left(u_{a}\right)\\ \overset{︷}{\left[\begin{array}{cccccc} k_{1}s\gamma_{1} & k_{1}s\gamma_{2} & 0 & 0 & 0 & 0\\ k_{1}c\gamma_{1} & k_{1}c\gamma_{2} & k_{1} & k_{1} & k_{1} & k_{1}\\ k_{1}c\gamma_{1}l-k_{2}s\gamma_{1} & -k_{1}c\gamma_{2}l+k_{2}s\gamma_{2} & k_{1}l\frac{1}{2} & -k_{1}l\frac{1}{2} & -k_{1}l\frac{1}{2} & k_{1}l\frac{1}{2}\\ 0 & 0 & k_{1}l\frac{\sqrt{3}}{2} & -k_{1}l\frac{\sqrt{3}}{2} & k_{1}l\frac{\sqrt{3}}{2} & -k_{1}l\frac{\sqrt{3}}{2}\\ -k_{1}s\gamma_{1}l-k_{2}c\gamma_{1} & +k_{1}s\gamma_{2}l+k_{2}c\gamma_{2} & -k_{2} & k_{2} & k_{2} & -k_{2} \end{array}\right]} \end{array}\begin{array}{c} u_{b}\\ \overset{︷}{\left[\begin{array}{c} \delta_{1}\\ \delta_{2}\\ \delta_{3}\\ \delta_{4}\\ \delta_{5}\\ \delta_{6} \end{array}\right]} \end{array}.$$

## 5. Simulation Setup and Experimental Results

This chapter presents the simulation setup and the results obtained for the proposed over-actuated HTR. The simulation setup includes development using PX4, Gazebo, and ROS, providing a versatile virtual environment to demonstrate the dynamic vehicle responses for the controls designed and the extended FCA method.

### 5.1. Simulation Setup

Virtual environments are a useful tool and good practice in the robot and algorithm development process before building and running code on physical robotic systems [19,58]. It is common to start by testing and evaluating theoretical methods in a simulator because the robots themselves are often complex, expensive, fragile, and scarce. These tools are frequently used in research due to their accessibility and provide a safe and low-cost space for testing and validating algorithms [59].

A widely used tool in the robotics community is Gazebo, which is designed to conduct software-in-the-loop simulations of UAVs. As an open-source simulator, Gazebo is intrinsically integrated with ROS, being a powerful tool that allows realistic scenarios to be created for flight simulations under different conditions, with physical properties such as wind, gravity, and collisions. For this work, the designed HTR was built in the Gazebo environment, along with its tiltable propulsion motors.

For this purpose, the PX4 Autopilot was employed. It is a popular open-source autopilot platform for controlling UAVs, covering a wide range of vehicle types, from fixed-wing and multi-rotor aircraft to water vehicles and submarines [60]. This platform offers a complete ecosystem of tools, including flight controllers and a wide array of supported sensors. As previously mentioned, all control loops and routines were implemented on it, ensuring the experiments were conducted under the most realistic conditions possible.

Lastly, ROS was used. It gives a robust suite of tools and libraries that facilitate the development process with code execution and communication with a distributed architecture. To communicate with the HTR and the high-level control logic, MAVROS serves as the middleware. Also, it is a ROS package and serves as a bridge between the PX4 and the ROS, allowing high-level commands such as SP and trajectory inputs to be transmitted to the PX4, which processes and executes these commands [61].

**Figure 8:**
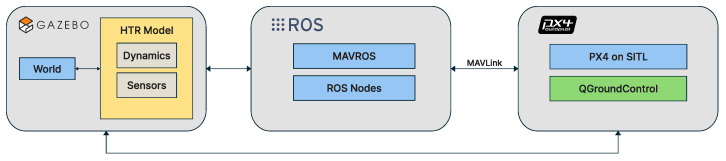
Representation of the communication architecture used in this work.

As illustrated in Figure 8, the communication architecture consists of three main components: Gazebo, PX4, and ROS.

It is also important to mention that the simulation was performed using an 11th generation 2.40 GHz Intel(R) Core i5-1135G7 device with 16 GB of RAM, a 64-bit Ubuntu 20.04 operating system, and an Intel(R) Iris(R) Xe Graphics.

### 5.2. Experimental Results

For the validation of the HTR proposed model, it is essential to analyze its performance in different scenarios to assess the accuracy and reliability of the responses. In this case, the HTR is evaluated in two open-field scenarios: the first involves a linear flight to validate the stability and control responses. The second scenario is a real-world PA application with different maneuvers.

In addition, to further validate the response of the HTR, it is compared with a traditional hexacopter model, specifically the Typhoon H480, chosen due to its compatibility with the PX4 Autopilot. The Typhoon H480 is simulated using its original dynamic parameters in Gazebo and default control gains in PX4, allowing for a direct comparison of flights.

#### 5.2.1. Scenario A

**Figure 9:**
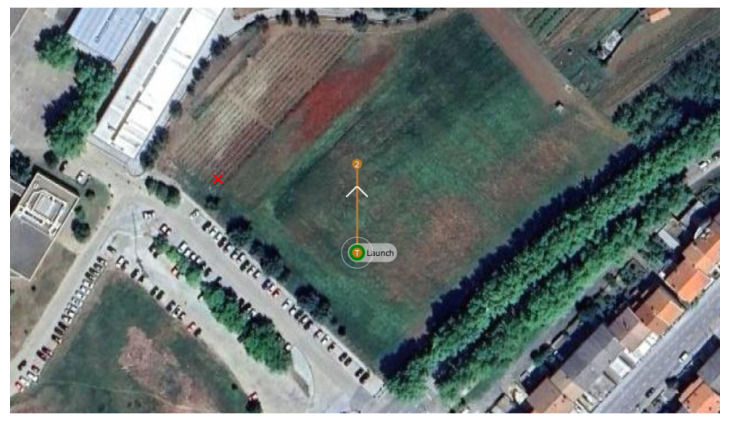
HTR flight path at the Polytechnic Institute of Bragança coordinates.

**Figure 10:**
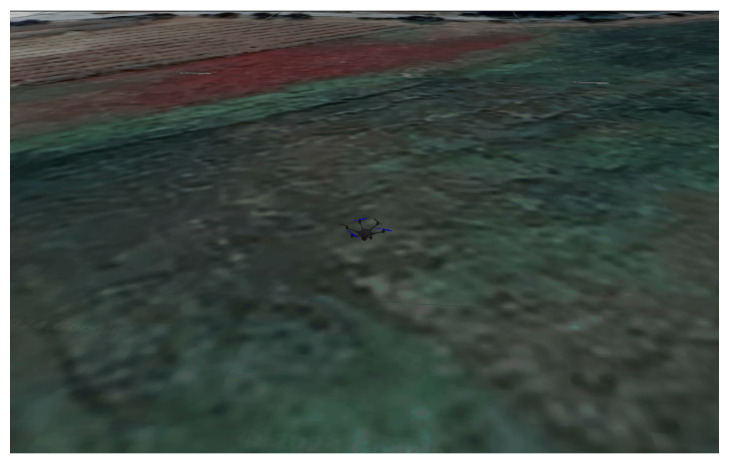
HTR flight in Gazebo.

For the first scenario, a linear path was generated, assuming the take off of the HTR, a straight-line flight (approximately 35 m), and landing. This flight was conducted at the Polytechnic Institute of Bragança (coordinates 41.796080° N, −6.767043° W), Portugal. Figure 9 and Figure 10 provide an aerial view from Google Maps and this location implemented in Gazebo world using real-world coordinates, respectively:

This location was selected for its safe and controlled environment, ensuring ideal conditions for the hexacopter tilt-rotor’s operation, guaranteeing an accurate assessment of the control system’s performance throughout the mission before its deployment in PA application. The open field is near a vineyard (indicated by a red ‘X’).

**Figure 11:**
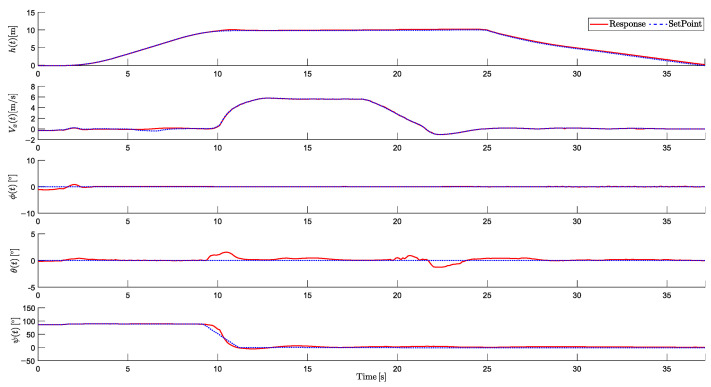
Controlled responses of altitude (*h*), velocity ($V_{x}$), roll (ϕ), pitch (θ), and yaw (ψ).

The altitude variable (*h*), linear velocity ($V_{x}$), and attitude angles (ϕ, θ, and ψ) are analyzed to validate the stability and precision of the proposed system. Additionally, the performance of the actuators is evaluated, including the control signals sent to the six motors and the two tilt rotors. Figure 11 shows the five controlled responses: altitude (*h*), linear velocity ($V_{x}$), roll (ϕ), pitch (θ), and yaw (ψ), respectively:

It is observed that the aircraft successfully reached the desired altitude dynamics. The HTR starts from a static position, and after taking off, it ascends to the desired altitude. Around t=10 s, the HTR initiates linear movement, and towards t=18 s, the velocity decreases as the landing process begins. Then, the landing is gradual, with a slightly longer duration to ensure enhanced safety. No overshoots were perceived.

Moreover, roll and yaw dynamics exhibited minimal variations, consistent with the planned trajectory conditions. Minor control oscillations were observed at the beginning of the roll response, which is related to the soil effect. However, the system maintained stability and quickly corrected minor disturbances. It is also important to highlight that the pitch response remained close to zero, with maximum deviations of ±1.2°. This is explained by the servomotors tilting angles when forward displacements are done.

**Figure 12:**
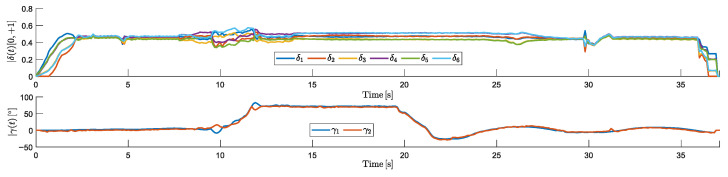
RCAs of HTR: responses from propulsion motors ($\delta_{i}$) and servomotors ($\gamma_{i}$).

Figure 12 shows the HTR’s RCA responses during the simulation:

The motors had the expected response throughout the simulated trajectory. Minor oscillations were observed when the aircraft reached the desired altitude during takeoff and when approaching the waypoint before landing. In addition, during the tilt rotor adjustments, motor oscillations were observed, because tilting the motors 1 and 2 degrees slightly reduces the total lift, requiring the remaining RCA to compensate. In response, the other motors temporarily increased their thrust to balance the lift loss.

It is also notable that during yaw maneuvers, the tilt directions were opposite. After reaching an altitude of 10 m, they tilted to approximately 70° and maintained this angle during the linear movement. Upon reaching the landing point, a negative tilt angle was applied to decelerate the HTR, ensuring a controlled reduction in velocity. Subsequently, the tilt rotors gradually retracted to their neutral positions, allowing a smooth landing.

**Figure 13:**
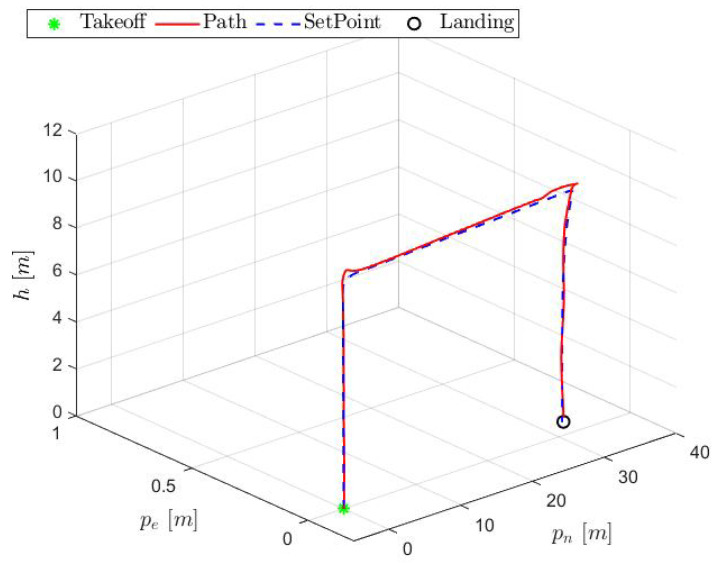
HTR three-dimensional path.

**Figure 14:**
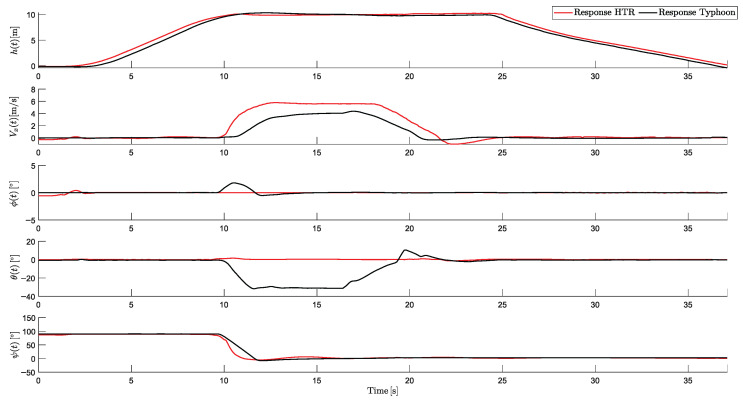
Comparative responses between HTR and traditional hexacopter (Typhoon H480).

Figure 13 illustrates the trajectory of the aircraft in a three-dimensional graphic:

As observed, the aircraft followed the planned path reasonable. Some metrics are presented in Table 2, taking the mean square error (MSE) into account:

To validate the responses of the HTR in this scenario, Figure 14 shows the comparative responses for the proposed HTR and this hexacopter flying in a traditional form (all six propulsion motors are in an upward direction):

As evident, the comparison highlights several key performance differences between the two aircraft. In terms of altitude, both vehicles demonstrated stable performance during takeoff, linear movement, and landing, with minimal oscillations. The mean altitude of the HTR in the linear movement was 10.01 m, compared to 9.95 m for the traditional one.

Additionally, the proposed HTR achieved a higher maximum linear velocity (5.6 m/s) compared to the traditional one (4.3 m/s), a difference of 32.97%. This result emphasizes the efficiency of the HTR in covering distances, maintaining 0 degrees of pitch throughout the trajectory, with angles of ±1.2°. In contrast, the traditional hexacopter relied on forward pitching (around −31°) to generate linear movement.

Regarding yaw control, the HTR exhibited a faster yaw response compared to the traditional model, enhancing its agility in maneuvers. This improved response time allows for more efficient displacements in this scenario compared to conventional aircraft.

This comparison also demonstrates the superior performance of the HTR, which completed the flight path with greater stability. These results validated the control implemented on the HTR and demonstrated its ability to keep zero pitch and high efficiency.

#### 5.2.2. Scenario B

For the second scenario, a trajectory was generated for a PA application focused on monitoring a vineyard.

The HTR performed a flight path at an altitude of 5 m, starting with take off, covering the plantation, and concluding with landing. This test was conducted over a vineyard at the Polytechnic Institute of Bragança (coordinates 41.796420° N, −6.767929° W), Portugal.

**Figure 15:**
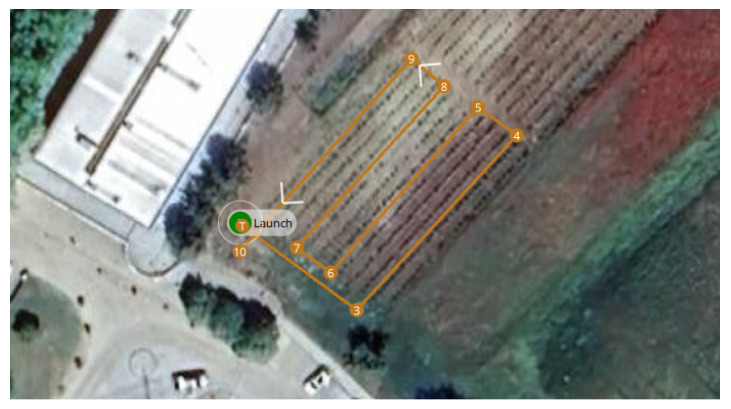
HTR second flight path at the Polytechnic Institute of Bragança coordinates.

**Figure 16:**
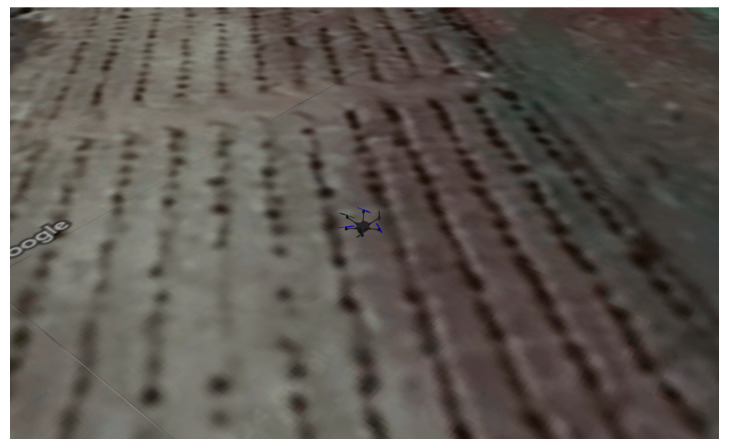
HTR second flight in Gazebo.

Figure 15 and Figure 16 display an aerial view from Google Maps and this location implemented in Gazebo world using real-world coordinates, respectively:

This location was selected to provide a real-world PA scenario, where precise navigation over crops is crucial for efficient operations such as disease and pest detection.

The analysis focuses on the same key variables: altitude (*h*), linear velocity ($V_{x}$), and the attitude angles (ϕ, θ, and ψ), which are essential for assessing the aircraft response in this PA environment.

**Figure 17:**
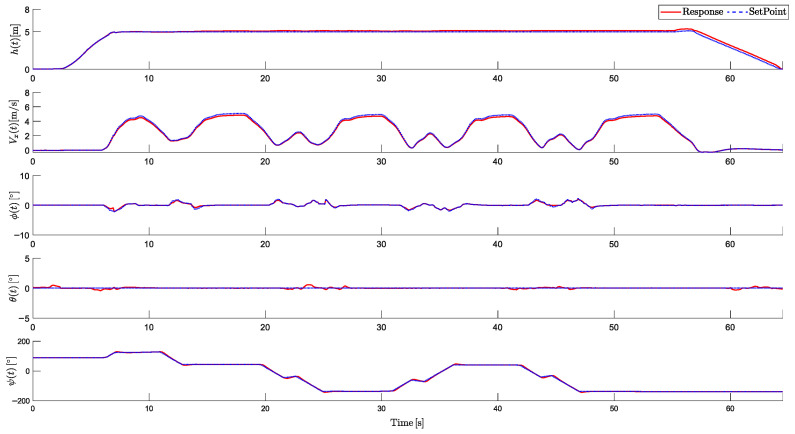
Controlled responses of altitude (*h*), velocity ($V_{x}$), roll (ϕ), pitch (θ), and yaw (ψ).

The actuator’s performance, including the control signals driven to the six propulsion motors and the two tilt rotors, is evaluated. Figure 17 illustrates the five controlled responses:

The graphics showed that the HTR demonstrated a response closely aligned with the SPs, with altitude stabilized at the desired value, indicating its capability to maintain precise navigation covering this area.

During the entire flight, the linear velocity follows the SP, with small oscillations due to controller corrections to adjust other dynamic variables. These deviations are expected, given the characteristics of the coupled variables in the system.

Regarding attitude variables, the HTR showed more changes in roll and yaw due to the curves present in the flight path, leading to increased fluctuations in these variables. The roll angles remained relatively low and close to the setpoint, indicating the HTR’s ability to perform maneuvers without significant deviations.

In contrast, the pitch remained close to zero, which aligns with the expected performance since the tilt rotors are responsible for this control function. Also, the yaw had more changes due to the HTR’s nose adjustments, which were required to follow the trajectory, confirming the efficiency of maintaining the correct heading throughout the flight.

**Figure 18:**
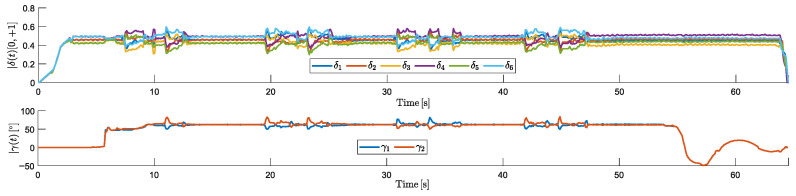
RCAs of HTR—Response from thrust motors ($\delta_{i}$) and servomotors ($\gamma_{i}$).

As a result, Figure 18 points out the RCA responses of the aircraft along the flight path:

This figure illustrates that the propulsion motors demonstrated the expected responses throughout the flight, with notable variations occurring during turn movements and tilt rotors movements. This happens because tilting the motors 1 and 2 degrees slightly reduces the total lift, requiring the remaining RCA to compensate. The other motors temporarily increased their thrust to balance the lift loss. Other small differences can be observed in both the takeoff and landing points. Throughout the flight, the six motors operated at an average of 54.39% of their maximum rotation capacity, ensuring stable performance during demanding maneuvers.

During straight-line segments, the tilt rotors maintained an angle of approximately 56° but kept the HTR with zero pitch for forward movement. In addition, during yaw maneuvers, opposing tilt angles were observed for servomotors 1 and 2. Throughout takeoff, the tilt rotors began in a neutral position and progressively increased their angles to achieve the desired velocity. Upon landing, the tilt rotors gradually transitioned to negative angles, reducing the HTR velocity as it approached the landing point.

**Figure 19:**
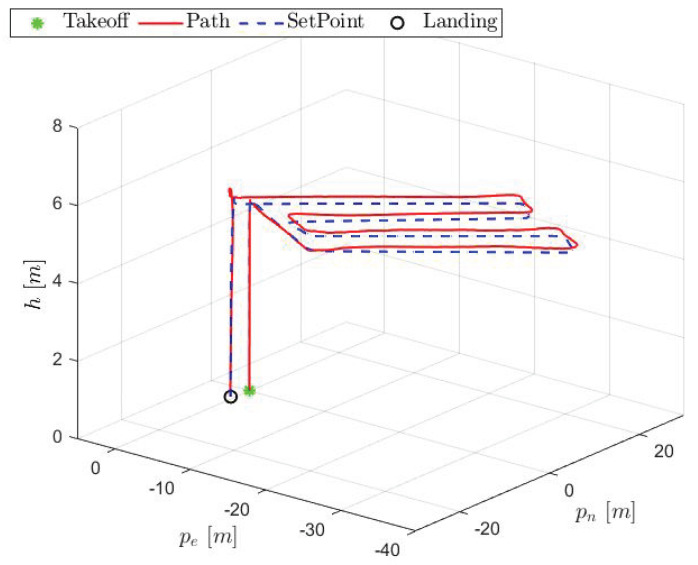
HTR three-dimensional path.

**Figure 20:**
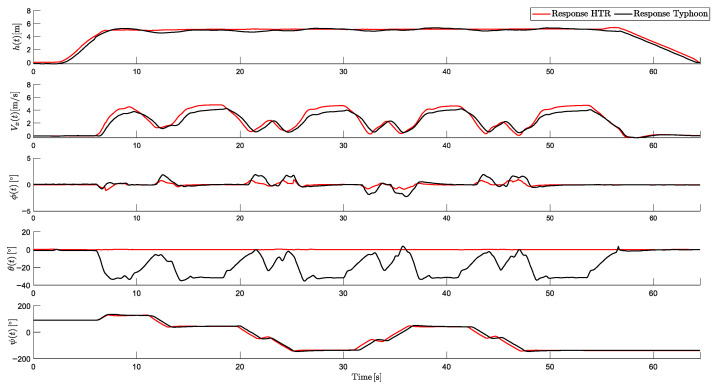
Comparative responses between HTR and traditional hexacopter (Typhoon H480).

Figure 19 depicts the aircraft trajectory in a three-dimensional graphic:

As could be seen, the aircraft tracked the planned path reasonably well. Some metrics are presented in Table 3, taking the MSE into account:

To validate the responses of the HTR in the scenario, Figure 20 illustrates the comparative responses for both aircrafts:

Several key points can be discussed from this comparison between the two hexacopters. In terms of altitude, both vehicles demonstrated stable performance during takeoff, linear movement, and landing, with minimal oscillations. The mean altitude of the HTR in linear movement was 5.1038 m. In contrast, the traditional hexacopter showed more variations, reaching a peak of 5.3227 m and 4.5419 m, with an altitude mean of 5.0080 m.

Regarding the linear velocity, the proposed HTR achieved a higher maximum linear velocity throughout the trajectory (4.8293 m/s) compared to the traditional hexacopter (4.2431 m/s). This is evident in the second graph, where it is observed a difference of 13.82% in the linear velocity.

In terms of attitude, the HTR exhibited a higher roll angle during the first orientation adjustment at t=6 s. In contrast, in the following instants, the traditional hexacopter displayed higher roll angles. The difference in pitch is particularly notable due to the HTR’s efficiency in covering distances, maintaining 0 degrees of pitch throughout the trajectory, with angles of ±0.5°. In contrast, the traditional hexacopter relied on forward pitching (around −30°) to generate linear movement. In terms of yaw, both hexacopters displayed similar responses in this scenario.

Overall, this comparison highlights that the HTR achieves enhanced performance, executing the proposed trajectory with more stability and adherence to the desired altitude. These results confirm the HTR’s control responses and demonstrate its effectiveness throughout trajectories. It ensures its capability to manage complex trajectories in PA applications.

## 6. Conclusions

According to the results obtained in this study, a stable and functional HTR was successfully designed and tested in a virtual environment. Through simulations, its behavior was validated, confirming the control algorithm’s implementation, and the effectiveness of the proposed control allocation method was demonstrated. The control system’s integration with the FCA technique proved essential for enhancing both stability and maneuverability.

The experimental results demonstrated that the HTR followed the proposed trajectories, achieving higher velocities of 5.6 m/s in Scenario A and 4.7 m/s in Scenario B. Altitude was stabilized, and the aircraft attitude remained aligned with the SPs. In particular, the pitch was consistently close to zero degrees, with small oscillations of ±1.2° in Scenario A and ±0.5° in Scenario B. The results confirm the tilt rotor’s ability to control the system.

In conclusion, this project successfully addressed all important aspects, from the HTR’s modeling to the robust control strategies development and implementation. It showed that this configuration provides a significant contribution to the autonomous operations in precision agriculture. The implementation and fine control loop tuning (as well as the FCA technique application) were essential to achieve the paper’s main goals.

### Future Works

For future work, following the successful implementation of the control method and the application of the control allocation technique proposed in the HTR, several promising avenues for future research emerge.

A potential direction is the physical construction and implementation of the HTR in open-field tests, in cooperation with other collaborative robots, to perform crop monitoring and pest detection missions with computer vision. In addition, future investigations could focus on the development and integration of novel control methodologies, including fault-tolerant control strategies.

Lastly, extending the FCA technique to other over-actuated vehicles, such as fixed-wing tilt rotors and boats, there is a good opportunity for this approach’s applicability. Investigating these extensions could provide knowledge about the adaptability and performance of control systems across different vehicle types.

## Figures and Tables

**Table 1:** P-PID controller gains obtained for HTR.

Variable	P		
$p_{n}$	$k_{p}^{n}=0.949$		
$p_{e}$	$k_{p}^{e}=0.949$		
*h*	$k_{p}^{h}=2.000$		
ϕ	$k_{p}^{\phi }=5.500$		
θ	$k_{p}^{\theta }=6.500$		
ψ	$k_{p}^{\psi }=5.270$		
	P	I	D
*u*	$k_{p}^{u}=3.000$	$k_{i}^{u}=0.440$	$k_{d}^{u}=0.100$
*v*	$k_{p}^{v}=3.000$	$k_{i}^{v}=0.440$	$k_{d}^{v}=0.100$
*w*	$k_{p}^{w}=4.000$	$k_{i}^{w}=2.000$	$k_{d}^{w}=0.100$
*p*	$k_{p}^{p}=0.138$	$k_{i}^{p}=0.181$	$k_{d}^{p}=0.002$
*q*	$k_{p}^{q}=0.142$	$k_{i}^{q}=0.189$	$k_{d}^{q}=0.003$
*r*	$k_{p}^{r}=0.181$	$k_{i}^{r}=0.186$	$k_{d}^{r}=0.003$

**Table 2:** MSE obtained for inertial controlled response from Figure 13.

Variable	$p_{n}$	$p_{e}$	*h*
MSE	0.01923	0.0085	0.0294

**Table 3:** MSE obtained for inertial controlled response from Figure 19.

Variable	$p_{n}$	$p_{e}$	*h*
MSE	0.0389	0.0536	0.0276

## Data Availability

The data used to support the findings of this study are available from the corresponding author upon request.
